# Vitamin D binding protein is lower in infertile patients compared to fertile controls: a case control study

**DOI:** 10.1186/s40738-017-0042-0

**Published:** 2017-10-10

**Authors:** Jason Franasiak, Sue Shapses, Wei Sun, Richard Scott, Xiangbing Wang

**Affiliations:** 1 Division of Reproductive Endocrinology, Department of Obstetrics Gynecology, Thomas Jefferson University, Philadelphia, Pennsylvania USA; 2 Reproductive Medicine Associates of New Jersey, Basking Ridge, 140 Allen Road, Basking Ridge, New Jersey 07920 USA; 3 Department of Nutritional Sciences, Rutgers University, New Brunswick, New Jersey USA; 4 Division of Endocrinology, Department of Medicine, Robert Wood Johnson Medical School, Rutgers University, New Brunswick, New Jersey USA

**Keywords:** Fertility, Menstrual cycle, Bioavailable vitamin D, Vitamin D binding protein

## Abstract

**Background:**

The importance of vitamin D in general health as well as in human reproductive success has been an area of focus. A better understanding of vitamin D metabolism, particularly vitamin D binding protein, is important when elucidating this relationship.

**Methods:**

This case control trial seeks to characterize vitamin D metabolism in infertile patients undergoing natural cycle IVF as compared to normally cycling premenopausal women with proven fertility matched for age and body mass index (BMI). A total of 68 subjects were examined; 39 were infertile premenopausal women and 29 were regularly cycling fertile controls. Their 25-hydroxy vitamin D (25OHD), vitamin D binding protein (DBP), and albumin were measured and free and bioavailable 25OHD calculated. Between group comparisons were conducted with an unpaired t-test. A stepwise regression using age, BMI, 25OHD, estradiol & albumin in the model were used to determine predictors of DBP.

**Results:**

Age, BMI, and total 25OHD did not differ between the two groups. However, vitamin D binding protein, free and bioavailable vitamin D were significantly different in the infertile patients as compared to the regularly cycling fertile controls (*p* < 0.01). Stepwise Regression using age, BMI, 25OHD, estradiol & albumin in the model showed that only albumin was a predictor of DBP (β-coefficient − 0.310; *p* = 0.01).

**Conclusion:**

The implications of lower vitamin D binding protein associated with infertility is not clear from this pilot study, and requires further study.

## Background

The 25-hydroxy vitamin D (25OHD) deficiency epidemic in the United States has prompted exploration into its relationship with many areas of human health and disease. It has subsequently been linked to many chronic diseases of the cardiovascular and metabolic systems [1–3] and recent work has evaluated its impact on human reproduction and obstetrical outcomes [4–6]. Given the potential for 25OHD to impact even early pregnancy, 25OHD status in patients undergoing infertility treatment has been of interest.

Investigation into this area using total 25OHD has resulted in conflicting findings. Some studies which evaluated follicular fluid correlated 25OHD levels to pregnancy outcomes [5]. Other studies evaluted serum 25OHD levels amongst different ethnicities as they related to pregnancy outcomes and found that levels correlated with outcomes in non-Hispanic whites, but no other ethnicites [6]. Another study charaterizing 25OHD status in patients undergoing euploid embryo transfer found no correlation between 25OHD status and pregnancy outcomes [7].

An issue with the 25OHD literature to date is a lack of full characterization of the differences in 25OHD metabolism parameters in infertile patients as compared to fertile controls. Further, the studies to date in this area have utilized assays for 25OHD that measure the total circulating 25OHD level. It has been suggested that a more comprehensive characterization of vitamin D status should include the measurement of bioavailable 25OHD, which requires assessment of 25OHD, vitamin D binding protein (DBP), and albumin [8, 9].

In the present study we seek to further characterize key components of 25OHD metabolism in infertile patients and fertile controls by measuring 25OHD, DBP, and albumin with subsequent calculation of free and bioavailable 25OHD followed by comparison of these parameters between groups.

## Methods

This was a retrospective study evaluating 25OHD parameters in a group of patients undergoing infertility treatment via modified natural cycle in vitro fertilization (IVF) as compared to patients who were premenopausal, normally cycling, and fertile controls. All study participants were informed of research participation and the study occurred under Institutional Review Board approval and written informed consent was obtained from the study participants.

### Patient population

The infertile patients in this study were those at a single IVF center who were undergoing a modified natural cycle IVF. In order to be included, patients had regular menstrual cycles of less than 39 days in duration. They were undergoing treatment due to unexplained infertility or fertility related to ovarian reserve. The patients were not on hormone therapy. These patients underwent routine medical screening that excluded thyroid, liver, and renal disease as determined by serum markers. These patients did not have recurrent pregnancy loss or autoimmune disease. As the patients were attempting conception, all were on a standard prenatal vitamin containing 200 mg of elemental calcium and 400 IU vitamin D3. No patients were taking additional vitamin D supplementation. Patients were counseled to ensure calcium intake (dietary plus supplements) meet the recommendations of 1000-1200 mg per day.

The fertile controls were all premenopausal women who had normal menstrual cycles and at least one prior live birth. The controls underwent a telephone screening and physical examination to ensure they met eligibility criteria. The fertile controls were originally recruited for a trial analyzing calcium and vitamin D metabolism [10]. Controls were asked to stop taking any dietary supplements for at least 4 weeks before the measurements were conducted and were given a standard daily multivitamin/mineral supplement containing 200 mg of elemental calcium and 400 IU vitamin D_3_. In addition, calcium intake (dietary plus supplements) was adjusted to meet the recommendations of 1000-1200 mg per day. Controls also had confirmation of normal thyroid, liver, and renal function as determined by history, physical examination, and thyroid function tests, complete metabolic panel, and complete blood count.

### Biochemical assessment

DBP levels in serum were determined using a commercial ELISA kit (Polyclonal antibodies, ALPCO, Salem, NH). All determinations of standards and samples were performed in duplicate. Levels of DBP were interpolated from a standard curve after measuring absorbance on an Elx808 plate reader using Gen5 data analysis software (BioTek Instruments, Inc). The intra- and inter-assay coefficients of variation (CV) for this assay were 5.0% and 12.7%, respectively. The published normal reference range for DBP levels [11] is 300–600 μg/ mL (30-60 mg/ dL). Serum samples were analyzed in duplicate for the following hormones: 25OHD (radioimmunoassay, RIA; DiaSorin, Stillwater, MN; CV < 12.5%). Estradiol was measured using an ultrasensitive estradiol radioimmunoassay (DSL, Webster, TX; CV < 8.9%).

#### Calculations

Free and bioavailable 25OHD were calculated using equations described by Vermeulen et al. [12]. The bioavailable hormone is the fraction that is both free and albumin-bound; thus, the fraction not bound to DBP. This method has been validated for calculation of free and bioavailable testosterone based on the measured total testosterone, sex-hormone binding globulin, and albumin along with the known binding-affinity constants for albumin and sex-hormone binding globulin. The formulas use the binding constants for 25OHD, DBP and albumin to calculate free and bioavailable 25OHD and are as follows: [Total] = concentration of 25OHD in g/mol ÷ 400.5 g/mol; [Alb] = serum albumin concentration in g/L ÷ 66,430 g/mol; [Total DBP] = concentration of serum DBP in g/L ÷ 58,000 g/mol; [D] = {[Total] – (K_alb_. [Alb] +1) ^.^ [D]} ÷ KDBP ÷ ([TotalDBP] – {[Total] – (K_alb_^.^ [Alb] +1) ^.^ [D]}); [Bio] = [D] + [D_alb_] = (Kalb ^.^ [Alb] + 1) ^.^ [D], where [Total] = Total 25OHD; [D] = free vitamin D; [Bio] = bioavailable vitamin D; K_alb_ = affinity constant between vitamin D and albumin = 6 × 10^5^ M^−1^; K_DBP_ = affinity constant between vitamin D and DBP = 0.7 × 10^9^ M^−1^.

### Statistical analysis

Descriptive data in terms of patient demographics and vitamin D assessment were summarized with mean and standard deviation for the entire group as well as for the fertile and infertile patients. Between group comparisons were conducted with an unpaired t-test. A stepwise regression using age, BMI, 25OHD, estradiol & albumin in the model were used to determine predictors of DBP. Statistical analysis was performed using SPSS (version 24, IBM Corp, Armonk, NY). Data are represented as mean ± SD. A *p* value <0.05 was considered statistically significant.

## Results

The distribution of vitamin D status indicates that the majority of women had 25OHD concentrations within normal range (Table 1). The mean age of the women was 38.0 ± 5.9 years, and the body mass index was 27.6 ± 4.4 kg/m2, and did not differ significantly between groups (Table 2). The patients’ self-reported ethnic breakdown was as follows: Black (9%), Asian (18%), and Caucasian (73%). The mean 25OHD level was 29.7 ± 9.3 ng/mL. Total serum 25OHD did not differ between the two groups with levels of 30.3 ± 9.8 ng/mL in the infertile group and 28.9 ± 8.7 ng/mL in the fertile controls (*p* = 0.57) (Table 2). However, when analyzing the infertile patients and fertile controls the level of DBP was 40.1 ± 12.5 mg/dL and 53.0 ± 24.0 mg/dL (*p* = 0.006), albumin was 5.2 ± 0.7 g/dL and 4.6 ± 0.2 g/dL (*p* < 0.001), calculated free 25OHD was 6.3 ± 2.9 pg/mL and 4.3 ± 1.8 pg/mL (*p* = 0.001), bioavailable 25OHD was 3.0 ± 1.4 ng/mL and 1.8 ± 0.8 ng/mL (p < 0.001), and estradiol was 116.0 ± 29.0 pg/mL and 45.3 ± 26.4 pg/mL (*p* < 0.01), respectively. Stepwise Regression using age, BMI, 25OHD, estradiol & albumin in the model showed that only albumin was a predictor of DBP (β-coefficient − 0.310; *p* = 0.01).

**Table 1:** Vitamin D status^a^

Status	Infertile (*n* = 39)	Fertile (*n* = 29)
12-20 ng/mL	12%	7%
	16.3 ± 2.7	17.9 ± 0.4
20-30 ng/mL	44%	68%
	25.4 ± 3.2	28.9 ± 8.7
>30 ng/mL	44%	24%
	39.1 ± 6.9	40.3 ± 10.7

^a^Institute of Medicine Guidelines for 25OHD status [Low levels < 50 nmol/L (20 ng/mL); Normal range: 50-125 nmol/L (20-50 ng/mL)]

**Table 2:** Characteristics of vitamin D metabolic parameters in premenopausal women

	Infertile (*n* = 39)	Fertile (*n* = 29)
Age (years)	37 ± 6	39 ± 6
BMI (kg/m2)	27.3 ± 5.4	27.9 ± 2.3
25OHD (ng/mL)	30.3 ± 9.8	28.9 ± 8.7
Albumin (g/dL)	5.2 ± 0.7*	4.6 ± 0.2
DBP (mg/dL)	40.1 ± 12.5*	53.0 ± 24.0
Free 25OHD (pg/mL)	6.3 ± 2.9*	4.3 ± 1.8
Bioavailable 25OHD (ng/mL)	3.0 ± 1.4*	1.8 ± 0.8
Estradiol (pg/mL)	116.0 ± 29.0*	45.3 ± 26.4

Values are mean ± SD. Differs from Fertile women, * *P* < 0.01

*Abbreviations*: *25OHD* 25-hydroxyvitamin D, *DBP* vitamin D binding protein

## Discussion

These data demonstrate there are significant differences in 25OHD metabolism between cohorts of infertile patients and fertile controls. Despite similar total 25OHD levels, DBP, free 25OHD and bioavailable 25OHD all differed between groups. This provides additional insight into the differences in health and disease when it comes to characterizing the infertile population.

It is important to note that both groups consisted of subjects that have normal menstrual cycles. It has been shown that timing of assessment during the follicular phase of the menstrual cycle when estrogen levels fluctuate most do not impact 25OHD metabolism parameters [13] which would suggest the variation seen is likely not due to synthetic differences driven by estradiol levels. It is likely some other factor drives the differences seen in DBP between the two groups, which ultimately lead to the differences seen in bioavailable and free 25OHD. The fact the DBP can have varied binding affinities adds a further layer of complexity [14].

It is known that 25OHD metabolism is influenced by a number of factors. Investigators have shown that age, BMI, ethnic background, as well as pre- and post-menopausal status affect the metabolism of 25OHD [11, 15, 16]. Here, age, BMI, and ethnicity did not differ between groups. Estrogen insufficiency, as is the case with postmenopausal women who have concentrations of about 13 pg/mL, have lower levels of 25OHD and DBP [17]. Further, exogenous estrogen increases DBP [18]. However, in regularly cycling women who experience a rise and fall of estradiol on a monthly basis, these levels will not decline to an appreciable degree [13]. In the current study, serum estradiol is within a normal range in both groups of women, and while it’s higher in the infertile women, it is likely above a threshold of estrogen in women cycling, whereby DBP is no longer affected. There is no evidence that the wide range of estradiol (15-350 pg/mL) found in premenopausal women influences DBP.

Anovulation is a cause of infertility and may be related to vitamin D metabolism since low 25OHD concentrations is associated with longer menstrual cycles [19] and low vitamin D intake may increase the risk of early menopause [20]. However, all of the patients with infertility in the current study had regular menstrual cycles. While these data do not offer an explanation why differences were seen between groups, it is possible that even though free and bioavailable 25OHD are higher in the infertile group, a low DBP plays a role in infertility. It is important to note this is a pilot study with a small sample size. These findings and this hypothesis should be confirmed in a larger cohort study with a large ethnic diversity.

## Conclusions

In summary, total 25OHD may be misleading when evaluating vitamin D status in infertility. The data presented show that while serum 25OHD does not differ between infertile and fertile women, DBP, free 25OHD, and bioavailable 25OHD are different in the infertile patients compared to fertile controls. Examining this in a larger dataset and determining the physiologic explanation for these findings is needed.

## Abbreviations

25OHD: 25-hydroxy vitamin D
BMI: Body mass index
DBP: Vitamin D binding protein
IVF: In vitro fertilization
